# Using pseudo-absence models to test for environmental selection in marine movement ecology: the importance of sample size and selection strength

**DOI:** 10.1186/s40462-022-00362-1

**Published:** 2022-12-29

**Authors:** Jérôme Pinti, Matthew Shatley, Aaron Carlisle, Barbara A. Block, Matthew J. Oliver

**Affiliations:** 1 College of Earth, Ocean, and Environment, University of Delaware, Lewes, DE 19958 USA; 2 Hopkins Marine Station, Biology Department, Stanford University, Pacific Grove, CA 93950 USA

**Keywords:** Movement ecology, Null models, Brownian motion, Random walks, Biotelemetry, Habitat selection, Environmental selection

## Abstract

**Background:**

Understanding the selection of environmental conditions by animals requires knowledge of where they are, but also of where they could have been. Presence data can be accurately estimated by direct sampling, sightings, or through electronic tag deployments. However, absence data are harder to determine because absences are challenging to measure in an uncontrolled setting. To address this problem, ecologists have developed different methods for generating pseudo-absence data relying on theoretical movement models. These null models represent the movement of environmentally naive individuals, creating a set of locations that animals could have been if they were not exhibiting environmental selection.

**Methods:**

Here, we use four different kinds of null animal movement models—Brownian motion, Lévy walks, Correlated random walks, and Joint correlated random walks to test the ability and power of each of these null movement models to serve as appropriate animal absence models. We use Kolmogorov-Smirnov tests to detect environmental selection using two data sets, one of simulated animal tracks biased towards warmer sea surface temperatures, and one of 57 observed blue shark tracks of unknown sea surface temperature selection.

**Results:**

The four different types of movement models showed minimal difference in the ability to serve as appropriate null models for environmental selection studies. Selection strength and sample size were more important in detecting true environmental selection. We show that this method can suffer from high false positive rates, especially in the case where animals are not selecting for specific environments. We provide estimates of test accuracy at different sample sizes and selection strengths to avoid false positives when using this method.

**Conclusion:**

We show how movement models can be used to generate pseudo-absences and test for habitat selection in marine organisms. While this approach efficiently detects environmental selection in marine organisms, it cannot detect the underlying mechanisms driving this selection.

**Supplementary Information:**

The online version contains supplementary material available at 10.1186/s40462-022-00362-1.

## Background

To understand the distribution of biodiversity in the ocean it is important to study how a particular species interacts with the physical environment [1]. The combination of biotelemetry (use of animal-borne electronic tags to remotely collect movement and behavioral, physiological, or environmental data and transmit these data to a receiver of some type) and ocean observations enables tracking individual organisms and their habitat use. This information provides the basis for understanding the autecology (e.g. species ecology) of tracked species, and therefore their management and conservation.

Since the 1990s, advances in aquatic biotelemetry have fundamentally changed marine movement ecology research. Researchers have now electronically tagged and tracked tens of thousands of marine organisms, including teleost and chondrichthyan fishes, marine reptiles, marine mammals, birds, and cephalopods [2–4]. These tracking technologies have provided researchers with a wealth of location and movement data of marine species. These observations include record migration distances [5], dive depth [6, 7] and duration [8], and the identification of unexpected life-history features, such as the discovery of the “white shark café” [9] or triennal migrations of soupfin sharks [10].

However, documenting observed movements alone does not always elucidate the underlying mechanisms that produced them. The movement patterns of an organism may resemble a theoretical movement model, but theoretical models do not capture the features or drivers of organismal movements. Quite the opposite, it is the sum of all external and internal factors and the animal’s individual capabilities that produced a track resembling that of a particular movement model [11]. Understanding the processes that lead to a specific movement is necessary to assess how organisms may react to environmental changes [12]. As such, understanding these linkages is fundamental in predicting what our future oceans will look like.

Electronic tags come in a variety of types (ARGOS, archival, GPS, and acoustic tags) and many do not record oceanographic conditions along with their estimated position. However, some provide depth and temperature profiles [13, 14] and others provide sophisticated data equivalent to oceanographic CTDs [15, 16]. For the tags that only provide positions, their locations can be combined with other environmental data sets from ocean observatories to investigate the underlying mechanisms and drivers of movement and to begin to understand the autecology of a species. For example, when location data are combined with satellite products, animal tracks can be used to investigate the drivers of shark vertical behaviors [13], the association between pelagic predators and mesoscale eddies [17], or the impact of oil spills on sensitive marine species [18]. At a finer spatial scale, high frequency radars can also help understand the link between Lagrangian oceanographic features and foraging [19]. These results can further be used to understand the conditions and mechanisms that shape animal movement, and understand how they might change in a changing climate [20].

Environmental selection is inferred through the difference between used environmental conditions and the available background conditions. These available background conditions can be determined using absence or pseudo-absence data [21, 22], or sometimes a step-selection approach which uses a step (connecting two sequential observations) as the unit of measurement [23–26]. Environmental selection is absent if the environmental conditions experienced by an organism have the same distribution as those experienced by an environmentally naive organism. While tags provide a variety of quantitative estimates of locations (presence of the organism), they cannot provide researchers with true absence data. The solution that is usually adopted is to use random walk models or other models (background sampling, kernel densities, other movement models) to simulate environmentally naive tracks that are used as distributions of pseudo-absences (“pseudo” because untagged animal distributions are unknown and may overlap with environmentally naive tracks) [17–19, 22, 27–30]. Different kinds of null models are appropriate in different settings [22, 31]. Without true absence data, using a large number of randomly generated pseudo-absences may be a reasonable approximation that still allows for ecological insight [32]. However, different “random” models follow different constraints which result in different pseudo-absence distributions.

Here, we examine four different movement models that generate pseudo-tracks which are then used as pseudo-absences for environmentally naive organisms, to determine if their choice as a null model has any impact on the ability to detect environmental selection. We start by describing four different possible pseudo-absence movement models: Brownian motion, Lévy flight, Correlated random walk and Joint correlated random walk. Each of these models relies on drawing two values at each time step: one for the step length, and one for the bearing. The difference between these is in how these two parameters are constrained by underlying movement theories. Then, we generate synthetic tracks (distinct from pseudo-tracks) that are biased towards high sea surface temperature (SST) to investigate how these generated pseudo-absences data can be used to test for environmental selection for SST. We then apply our method to 57 blue sharks tagged with Wildlife Computer SPOT tags in the North-East Pacific. Finally, we discuss important caveats for the interpretation of results using this method, including sample size, seasonality, and error rates.

## Methods

### Data used in this study

**Fig. 1:**
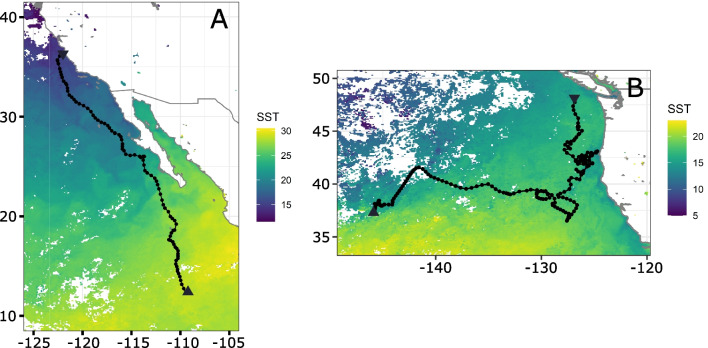
Observed blue shark tracks #160401201 (**A**) and #160700501 (**B**), overlaid on top of SST field for tag deployment day, November 15th 2004 (**A**) and July 25th 2007. Triangle pointing down marks tag deployment, triangle pointing up marks tag pop-up location

Blue shark (*Prionace glauca*) tracking data used in the analyses were collected during the Tagging of Pacific Predators (TOPP) project [3], and processed in a state-space model [33] resulting in a uniform 1 day temporal resolution across the dataset. The 57 blue sharks were tagged with Wildlife Computer SPOT tags between June 26th 2002 and November 1st 2009. Track lengths are between 8 and 349 days long (median of 88 days), resulting in 5572 daily blue shark position estimates in the North Pacific Ocean, with a median step length of 32.7 km. The tracks used as illustrative example (Fig. 1) are 90 and 207 days long, from November 15th 2004 to February 12th 2005 (tag #160401201) and from July 25th 2007 to February 16th 2008 (tag #160700501). Median step lengths of these tracks are 37.3 km and 29.3 km, respectively.

**Fig. 2:**
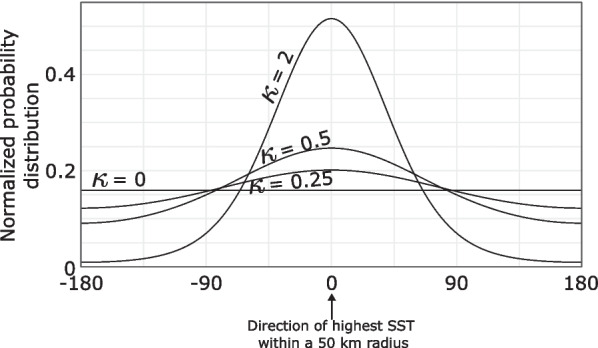
Von Mises distribution of relative bearing for movement models biased toward the highest temperature within a 50km radius for different values of $\kappa$

In addition to these directly observed tracks, synthetic tracks programmed to actively select for warmer sea surface temperature (SST) were simulated. These simulated presence tracks will be referred to as synthetic tracks (as opposed to simulated pseudo-absence tracks presented in the following section). These tracks were 80 days long to reflect a “typical” blue shark track length in the TOPP data set. For these synthetic tracks, step length was fixed at 50 km per day, and turning angle was generated following a Von Mises circular probability distribution [34] centred at the relative bearing corresponding to that of the highest SST within a 50 km radius (Fig. 2). The stronger the selection, the more likely the simulated animal will travel in the direction of the highest SST. $\kappa$ is the concentration (analogous to a standard deviation of a normal distribution) of the Von Mises distribution. $\kappa = 0$ means that organisms do not select for specific temperatures, while higher $\kappa$ values mean stronger selection towards higher temperatures. Details of biased random walk simulation are provided in supplementary information 1 for visual inspection of the effects of different values of $\kappa$. These synthetic tracks serve as an alternate “presence” with a known selection strength. For each value of $\kappa$ tested (0, 0.25, 0.5, 0.75, 1, 2, 5, 10, 20), 100 synthetic tracks were created.

The environmental fields in this study are satellite-derived sea surface temperature (SST) from MODIS-Aqua [35]. SST fields have a native 9 km spatial resolution. The native time step was 1 day, but each day consisted of a backward rolling average of the previous 8 days to increase data coverage.

### Null movement model generation

#### Step length and turning angle of observed blue shark tracks

To make sure our movement models had similar movement characteristics to observed blue shark tracks, we estimated the distributions of the step lengths and turning angles of the 57 observed tracks. We used these distributions as a basis for constructing our four random movement models. The tracks were processed by a state-space model [33] that regularizes the temporal distribution of positions and interpolates the track when raw ARGOS location estimates are missing. This interpolation means that the turning angle distributions used here are probably straighter that that of the real shark path, which would slightly bias the creation of null movement models. However, this is not an issue as we are testing different kinds of null models, with different turning angle distributions (uniform or following the turning angle of the tracks—the turning angle of the real shark path being somewhere in between).

**Fig. 3:**
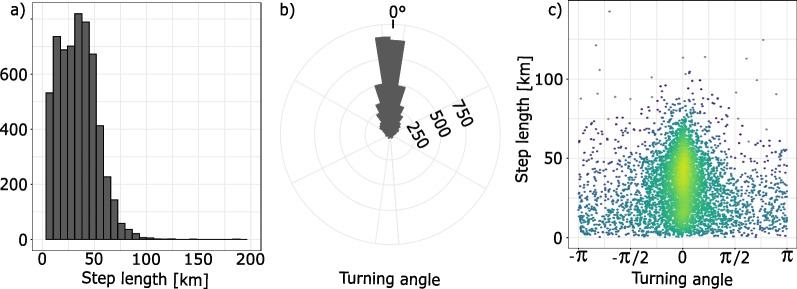
Characteristics of observed blue shark tracks. **a** Step length distribution (median 32.7 km), **b** turning angle distribution (median 0.0$^\circ$), **c** step length as a function of turning angle. Color of panel c mimics the density of data points in the turning angle-step length space: yellower areas have a higher density of data points

As the data are not normally distributed, we log-transformed the initial data to compute means and standard deviation of the tracks (Fig. 3). To account for potential seasonal biases, step length parameters were computed monthly.

#### Brownian motion

Brownian motion mimics the random motion of particles colliding in a fluid [36]. In movement ecology, it is often used to model the diffusion of animals moving without *a priori* selection [19, 37, 38].

At each time step, the organism moves a distance $\Delta l$ in a random direction. This means that the turning angle is drawn from a uniform distribution bound between $-180$ and $180^{\circ }$. We drew $\Delta l$ from a Gaussian distribution with mean and standard deviation computed from the observed data.

**Fig. 4:**
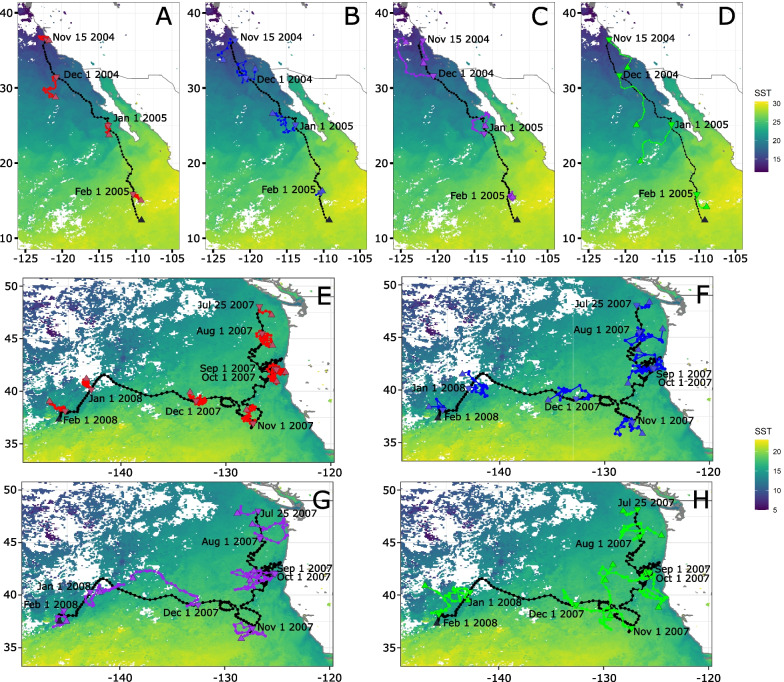
Pseudo-absence tracks (colors) and observed track (black). **A**–**D** Presence and pseudo-absence for track 160401201. **E**–**H** Presence and pseudo-absence for track 160700501. Tracks are overlaid on top of SST field for tag deployment day (November 15th 2004 for **A**–**D**, July 25th 2007 for **E**-**H**). Triangle pointing down marks track beginning (either true beginning or beginning of month), triangle pointing up marks track end (either true end or end of month), and dates are the dates corresponding to the beginning of tracks (either true beginning or beginning of month). **A**, **E** Brownian motion track in red, **B**, **F** Lévy flight in blue, **C**,**G** Correlated Random Walk in purple, **D**, **H** Joint Correlated Random Walk in green

These simulated tracks are a random sampling of the environment around the initial point according to the constraints of Brownian Motion (Fig. 4A and E). In Brownian Motion, diffusivity scales with the square root of time. Therefore, the movement is not bounded and it will eventually cross any circle of finite radius centred around the initial position given enough time. This is also one of the great advantages of Brownian motion. As such, to create a pseudo-absence track with unevenly spaced data (in case of e.g. missing presence data), the time step can just be multiplied by $\sqrt{\frac{\Delta t_{new}}{\Delta t_{ref}}}$, with $\Delta t_{new}$ the time step required and $\Delta t_{ref}$ the reference time step.

However, because Brownian motion is isotropic (meaning that at each time step the organism is equally likely to go in any direction) it may not sufficiently approximate wide-ranging or migrating animals. One possibility is to model the movement as a Brownian bridge, that is a Brownian motion whose initial and final positions are fixed [39]. However, as Brownian bridges may also produce tracks that are not believable representations under some conditions (For example, long distance central place foragers that have departure and arrival points close to each other), we are not using this method here. A pragmatic solution to this problem without applying a different movement model class with different constraints is to reset the Brownian track simulation to the actual animal position after a fixed period of time. In this study, the environmental sampling by the Brownian track is the aggregation of month-long Brownian tracks each starting at an actual organism location on the 1st of each month. This way, a simulated track would be constrained to the area around the real track and could still be reasonably considered to be in the same environment. For similar reasons, we will apply the same resampling strategy with the other movement models. To our knowledge, this is the first time that null movement tracks are created by restarting the track after fixed time intervals—except in the case of step selection functions, where each location serves as a new start point for the next step. The influence of the restarting time step on the resulting tracks and ensuing test results is further developed in supplementary material 1.

#### Lévy flight

Another type of isotropic random walk is Lévy flight (or Lévy walk). Lévy flights are a mix of many short foraging steps and fewer long relocation steps, hypothesised to mimic the optimal foraging of animals in a patchy environment [40, 41]. Lévy flights have spurred the interest of many researchers and have been reported across a range of marine species, such as wandering albatrosses [42], pelagic fishes [43], and microzooplankton [44]. However, whether or not Lévy flights is a true movement model, or an emergent pattern dependent on other underlying constraints is subject to controversy [11, 45, 46]. Despite this, Lévy flights might still be a useful null movement model, as they often have a structure similar to that observed in animal data.

Lévy flights are characterised by a uniformly distributed turning angle, but the step length distribution is heavy-tailed and follows a power law: $p(l) \sim l^{-\mu }$, with $1<\mu \le 3$ [47]. *l* is bounded by a minimum step length $l_{min}$ that is strictly positive (here taken equal to 1m). $\mu$ has to be strictly greater than 1, otherwise the equality $\int _{l_{min}}^{\inf } p(l) dl = 1$ cannot be satisfied. Conversely, the condition $\mu \le 3$ is needed to ensure infinite variance: the contrary would simulate Brownian-like motion [46].

A direct consequence of this choice is that the step length variance is infinite [11, 38], and that the probability of extremely long jumps *l* decreases with probability $l^{1-\mu }$—but remains possible.

A pragmatic solution to prevent extreme step lengths is to simulate truncated Lévy flights, i.e. Lévy flights with a finite maximum step length $l_{max}$. This implies a finite step length variance and thus relaxes the condition on $\mu$ that now only needs to be positive. The step length probability distribution is then:

1 $$\begin{aligned} p(l) = \left\{ \begin{array}{ll} 0 &{} \hbox {if }l<l_{min} \\ \frac{1-\mu }{l_{max}^{1-\mu }-l_{min}^{1-\mu }} l^{-\mu }&{} \hbox {if }l_{min}<l<l_{max} \\ 0 &{} \hbox {if }l_{max}<l \end{array} \right. \end{aligned}$$

if $$\mu \ne 1$$, and

2 $$\begin{aligned} p(l) = \left\{ \begin{array}{ll} 0 &{} \hbox {if }l<l_{min} \\ \frac{1}{\ln (l_{max})-\ln (l_{min})} l^{-\mu }&{} \hbox {if }l_{min}<l<l_{max} \\ 0 &{} \hbox {if }l_{max}<l \end{array} \right. \end{aligned}$$

if $$\mu = 1$$.

Similarly to Brownian motion, the simulated Lévy tracks (Fig. 4B and F) allow for a random sampling of the environment but with different constraints. The presence of occasional longer time steps results in a qualitatively wider diffusion than Brownian motion.

#### Correlated random walk

Brownian motions and Lévy flights are isotropic movements. However, in nature, most animals do not exhibit isotropic movements but tend to persist in their direction of movement (at least partly because of the cephalo-caudal polarization and bilateral symmetry of many animals [48]). Correlated random walk (CRW) movement models account for this persistent directionality, where each turning angle is correlated to the previous one [38]. CRW movement models have been shown to have similar properties as locally moving animals [49], and some marine animals have been shown to display movement patterns similar to CRW (e.g., [31]).

The step length of correlated random walks is generated from a distribution resulting in positive step lengths, such as an exponential or a gamma distribution. The turning angle of the animal is simulated with circular distributions that account for a preferential directionality, such as Von Mises or wrapped Cauchy distributions [34]. Determining which step length and turning angle distribution to use can be circumvented by using the observed distribution of step length and turning angles to simulate a CRW movement model (Fig. 3a and b).

Resulting tracks look more similar to actual animal movements than Brownian motion or Lévy walks (Fig. 4C). Dividing step length and turning angle simulation by months allows for a better representation of seasonal changes in behaviors. In the illustrated track, months with the strongest migration behavior (November to January) show a much straighter track than months with a more resident-like type of movement (August-October, Fig. 4C and G).

#### Joint correlated random walk

CRWs take into account the polarization of the animal, but treat step lengths and turning angles as separate, independent variables. This decouples step lengths from turning angles. In reality, step lengths and turning angles are often related, with longer step lengths having smaller turning angles, especially during pelagic migrations [50]. A way to consider this correlation is to draw from a single, two-dimensional empirical probability distribution at each time step in the turning angle—step length space (Fig. 3c). This effectively means that long time steps with large turning angles have a low probability, thus increasing the similitude between observed and simulated tracks while still having an environmentally naive model (Fig. 4D and H). If multivariate distributions that allow for correlated step lengths and turn angles need to be fit (e.g. to avoid using empirical distributions), the method of copulae can be used [51].

### Hypothesis testing

**Fig. 5:**
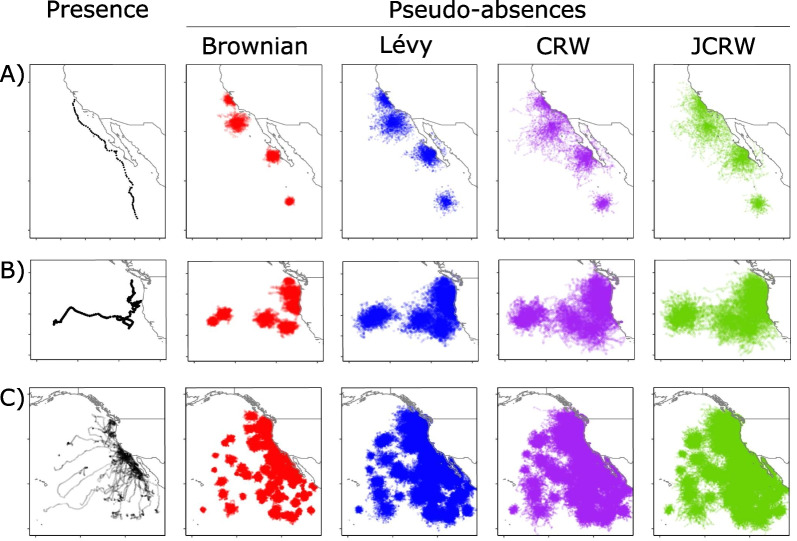
Maps of presence and pseudo-absences for blue shark #160401201 (**A**), blue shark #160700501 (**B**), and for all tagged blue sharks (**C**), respectively. The columns are for the tagged animals (presence), Brownian motion, Lévy walk, correlated random walk, and joint correlated random walk respectively

For each track (synthetic and observed), 100 tracks each of Brownian motion, Lévy walks, Correlated random walks, and Joint correlated random walks were simulated (Fig. 5). We used these to examine the ability of this approach to detect prescribed environmental selection in simulated pelagic animals and to determine whether or not observed blue shark tracks show evidence of SST selection (Table 1).

Each position (presence, simulated presence, and pseudo absence (Table 1)) was matched to its corresponding SST (sea surface temperature) value, derived from MODIS-AQUA SST fields (Fig. 1). For synthetic tracks, the position of the (synthetic) organism is assumed to be perfectly known, so only the point value at that location was used. However, for real organisms, position is associated with a confidence interval based on error associated with position estimates. For real animals, SST value is computed assuming that the error follows a 2D Gaussian distribution around the position estimate (i.e. the closer the observation is to the estimated location, the stronger the weight of this observation) with a standard deviation equal to 25% of the 95% confidence interval. Pseudo absences of real animals are assumed to follow the same structure, and uncertainties around each pseudo absence is the uncertainty of its corresponding presence point.

**Fig. 6:**
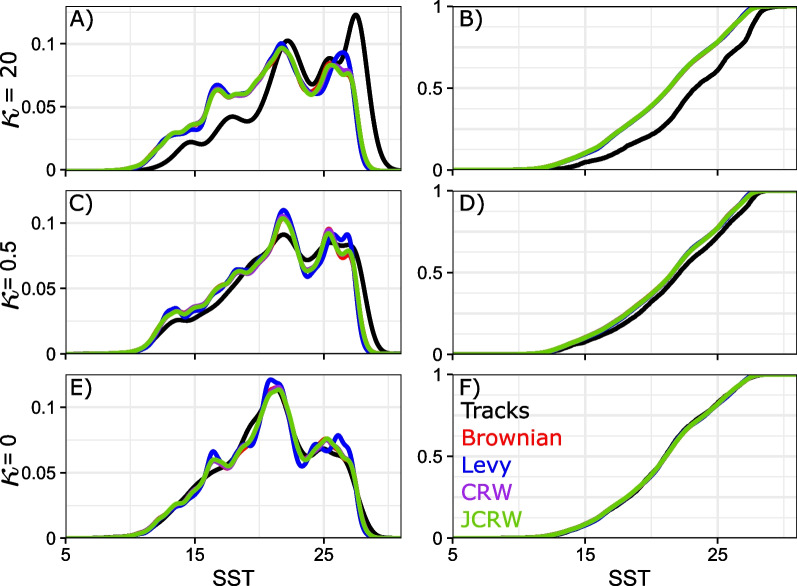
Density distribution (**A**, **C**, **E**) and cumulative distribution (**B**, **D**, **F**) of SST at synthetic presence and pseudo-absence points, for presence tracks strongly biased towards high temperatures (**A** and **B**), presence tracks weakly biased towards high temperatures (**C** and **D**), and presence tracks with no temperature bias (**E** and **F**)

The resulting SST distributions are pictured in Fig. 6 for synthetic tracks, and figure S8 for blue sharks. To test if there is evidence of selection in SST, we perform one-sided Kolmogorov-Smirnov tests (ks.tests in the R stats package, R: A language and environment for statistical computing, R Development Core Team). Kolmogorov-Smirnov tests (KS tests) are non-parametric tests comparing two cumulative distributions. The KS test statistic *D* is the maximum distance between the two cumulative distributions. In one-sided tests, we test the null hypothesis that the cumulative distribution of SST for presences is “not less than” (or “not greater than”) pseudo-absences SST distributions. For the “not less than test”, if the null hypothesis is rejected it means that the presence cumulative distribution function is below that of the pseudo-absence. This means that the temperature distributions are shifted toward higher temperatures, and that animals select for warmer waters compared to environmentally naive organisms. Conversely, for the “greater than” test, it means that animals select for colder waters than environmentally naive organisms). Throughout this manuscript and unless specified otherwise, tests are assumed significant for p-values $\le$ 0.05.

**Table 1:** Summary of the presence and pseudo-absence data used in this study, and their associated research question

Presence	Pseudo-absence	Research question
Synthetic tracks (Biased Random Walk, $\kappa = 20$)	Brownian motion, Lévy walk, CRW & JCRW	Can this method detect strong environmental selection?
...	...	...
Synthetic tracks (Biased Random Walk, $\kappa = 0.25$)	Brownian motion, Lévy walk, CRW & JCRW	Can this method detect weak environmental selection?
Synthetic tracks (Biased Random Walk, $\kappa = 0$)	Brownian motion, Lévy walk, CRW & JCRW	Can this method detect the absence of environmental selection?
Blue shark tracks	Brownian motion, Lévy walk, CRW & JCRW	Do blue sharks select for particular SST conditions?

## Results

### Synthetic tracks

**Fig. 7:**
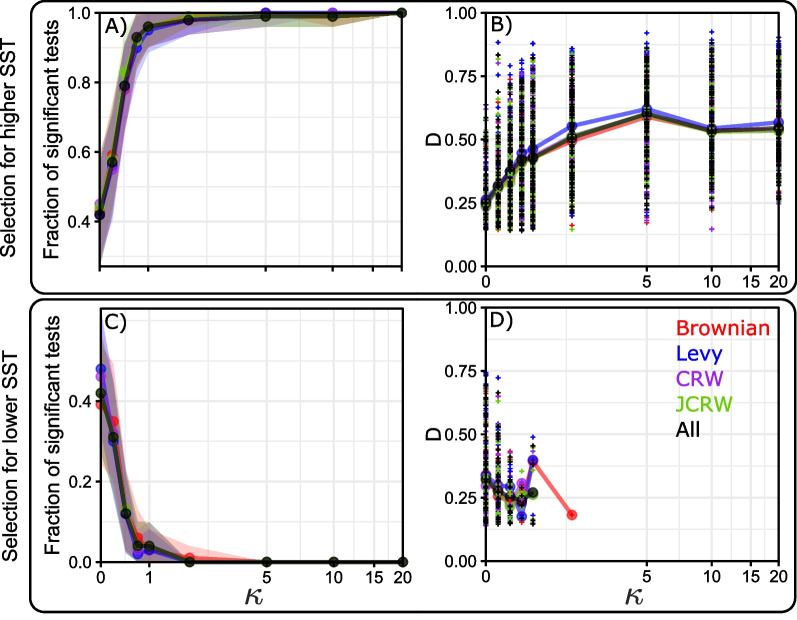
Fraction of significant KS tests with bootstrapped 95% confidence intervals (**A**, **C**), and test statistic *D* for statistically significant results (**B**, **D**) as a function of $\kappa$. Top (**A**, **B**): Tests for higher SST selection. Bottom (**C**, **D**): Tests with lower SST selection

We tested each 80-day synthetic track for environmental selection. For $\kappa > 2$, the results are consistent with what we should expect: nearly all tests for higher SST selection are significant and nearly no test for lower SST selection is significant (Fig. 7). However, when the strength of selection decreases ($\kappa < 2$), the fraction of false-positive (significant tests for lower SST selection) and false-negative (non-significant tests for higher SST selection) tests significantly increases. For $\kappa = 0$ when no test should yield significant result, approx. 40 % of all tracks show signs of selection for warmer temperatures, and 40% for colder temperatures when testing one 80-day track at a time. All these rates appear to be similar across the range of null models tested (Brownian motion, Lévy walks, Correlated Random walks, Joint Correlated Random walks, or the aggregation of these four). The strength of the selection (test statistic *D*) appears to vary widely for all values of $\kappa$, with a slight increase in strength for tests for higher SST selection as synthetic tracks select more and more strongly warmer temperatures (Fig. 7B). Interestingly, when there is no selection, CRW and JD pseudo-absences are generated following the same set of rules as presence data, but false-positive results still arise at low sample sizes.

**Fig. 8:**
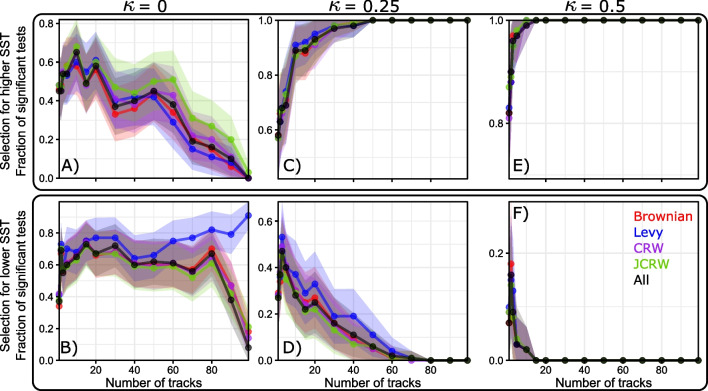
Fraction of significant test results as a function of the number of tracks considered for different values of $\kappa$, along with bootstrapped 95% confidence intervals. Top (**A**, **C**, **E**): Tests for higher SST selection. Bottom (**B**, **D**, **F**): Tests for lower SST selection

**Fig. 9:**
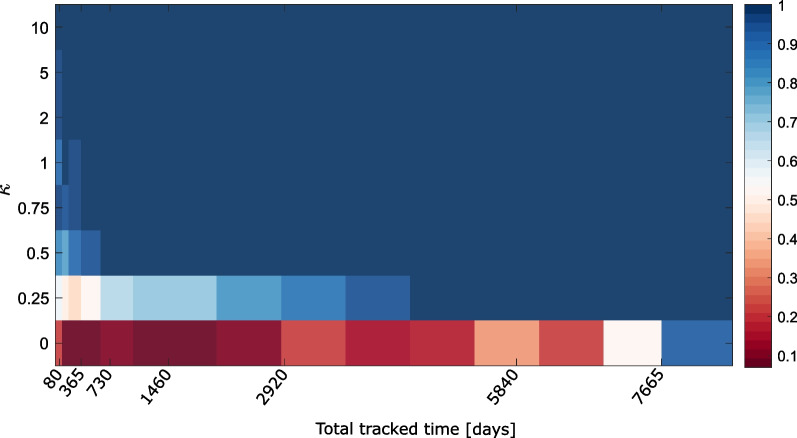
Fraction of correct test results (for both tests) as a function of dataset size and strength of temperature selection

One of the parameters that influence the most test results is sample size. Here, all synthetic tracks are 80 days long, which is a “typical” track length of a blue shark from the TOPP data set, but in reality, this limited track duration makes it difficult to avoid both false positive and false negative results [52]. One way to solve this issue is to aggregate multiple tracks together to increase the ability of KS tests to detect the presence of environmental selection. We investigate the fraction of significant results depending on the number of synthetic 80-day tracks aggregated together (Fig. 8). For relatively weak temperature selection ($\kappa = 0.5$), we get to 100% correct predictions as soon as we aggregate 1200 days (15 80-day tracks) of track time. For very weak temperature selection ($\kappa = 0.25$), perfect assessment of selection is only achieved with more than 17.5 years of total tracking time (or 80 80-day tracks). The hardest case to detect is when there is no environmental selection, as false positive results are still present even when the entire data set (100 tracks or 8000 days of track data) is aggregated. More concerning, tests performed with Lévy walks as pseudo absence data seem to converge towards 100% significant results for the lower SST selection alternative, meaning that acquiring more data would incorrectly predict that these organisms (not selecting for temperature in any way) select for colder temperatures. Fraction of correct (i.e. non-significant tests for lower SST selection and significant tests for higher SST selection) results as a function of selection strength and total tracked time are summarized in Fig. 9. Predictably, the stronger the selection, the fewer data needed to get correct results. For weak selections, it would appear that $\sim$ 4 years of daily data can already lead to acceptable results, whereas the no selection case would require more than 20 years worth of daily data to be confident that no selection exists. This means that, with a limited sample size of observed tracks, detecting environmental selection is difficult.

**Fig. 10:**
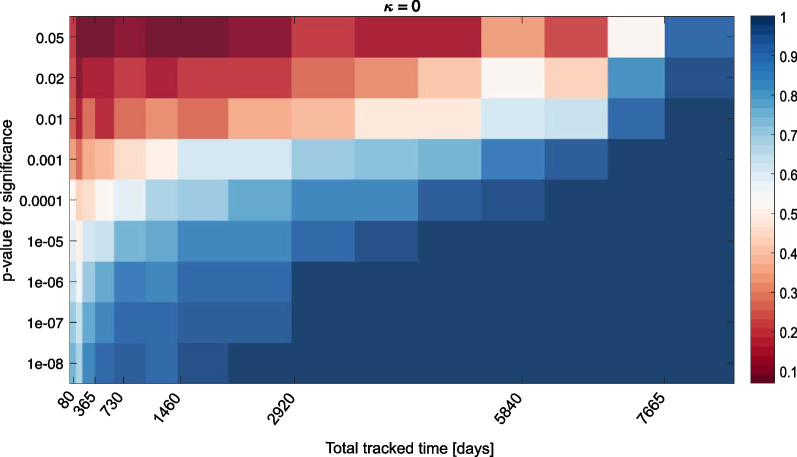
Fraction of correct test results as a function of dataset size and *p* value threshold for significance, when $\kappa = 0$ (no SST selection). Red colors are false-positive results, whereas blue colors display correct results for both tests (no selection)

Decreasing the p-value threshold for significance decreases the rate of false positives. Therefore, we can investigate how the rate of false-positivity increases when the p-value threshold changes (Fig. 10). As expected, when the threshold for significance is reduced, the fraction of tracks correctly categorized as “non-selecting” for higher or lower SST increases. With $p = 10^{-6}$, 100% of the tests yield correct results as soon as we have more than 8 years of track time (assuming one location per day per track). With $p = 10^{-6}$, this success rate is attained with 5 years of tracking data. Similarly, we can investigate the rate of false-positive and false-negative results for tracks with a weak SST selection (Additional file 1: Figs. S10 and S11). As expected, rate of false negative increases when the threshold for significance is decreased, but the fraction of false positive results is very low as soon as the p-value threshold for significance is below $10^{-6}$. As such, selection results with $p< 10^{-6}$ can be considered reliable with as little as two years worth of tracking data (even with a weak selection), while non-selection results are reliable with $p< 10^{-7}$ and more than 8 years of data.

### Temperature selection in blue sharks tracked with ARGOS SPOT tags

**Fig. 11:**
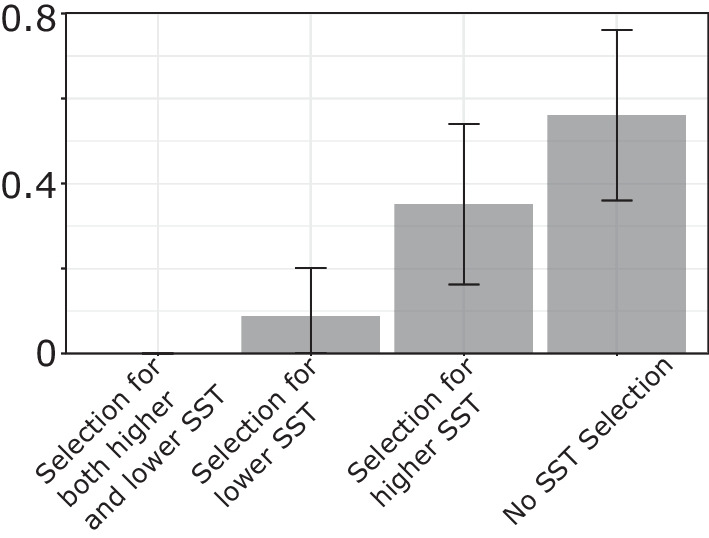
Fraction of blue shark tracks with both one-sided KS tests significant (0/57), one significant KS test (5/57 and 20/57), and no significant test (32/57), and associated bootstrapped 95% confidence interval

Testing SST selection in individual blue sharks tracked with ARGOS SPOT tags yields contrasting results (Fig. 11). More than 55 % of tracks have no significant result, which could indicate that blue sharks do not select for specific SST, or that tracks are not long enough to get significant results (see Figs. 9 and 10). Around 8% of blue sharks seem to select for colder SST, and 35 % seem to select for warmer SST. Further, we investigate the six longest tracks available for which we have more than 200 days of data (Additional file 1: Fig. S9). Out of these six tracks, 5 (all but track #160400601) reveal significant selection for warmer SST, while the remaining one does not reveal any selection (for warmer or colder SST).

A way to increase confidence in results is to increase sample size, which can be done by aggregating tracks together. However, as temperature selection can be seasonal, we aggregate tracks at different time scales—from a monthly scale to a yearly scale. Tables 2 and 3 summarize the KS test results in the case where pseudo absences are all null models taken together.Tables 2 and 3 were not color-coded based on *p* value only, but based on p-value and sample size, following our analysis of the previous section. A green cell means that selection was detected with more than 90 % confidence that it was not a false-positive result, and a red cell means that no selection could be confidently assessed. KS test results for all specific null models as absence data are given in supplementary information 2. There is little variation in test significance among the different null movement models.

At quarterly (or longer) time scales, all tests for higher temperature selection are significant, and no tests for lower temperature selection is significant, strongly suggesting that blue sharks tend, overall, to select for warmer temperatures. At finer scales, however, there are differences and no selection could be detected between February and July and in September–October. These seasonal differences might be due to seasonal variations in blue shark behavior (i.e. migrations), but this is beyond the scope of this paper and these results are not reliable given our findings from the previous section and the limited number of observations for each month.

**Table 2:**
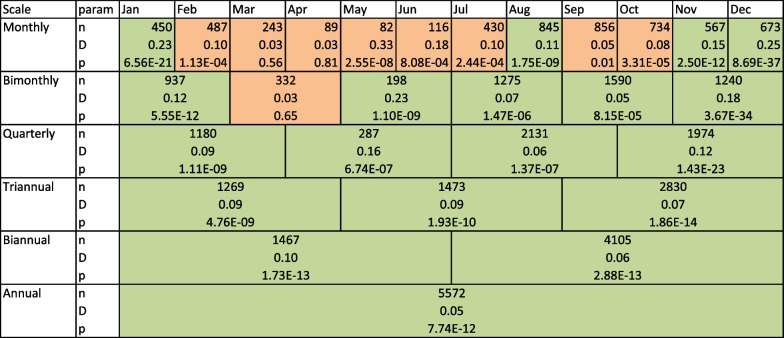
Summary of KS test results (selection for higher SST) for blue sharks at different aggregating scales

*n* refers to the sample size of observed blue sharks, *D* to the test statistic, and *p* to the p-value of the test. Cells are color-coded following the confidence in the test being correct, following the analysis of the previous section

**Table 3:**
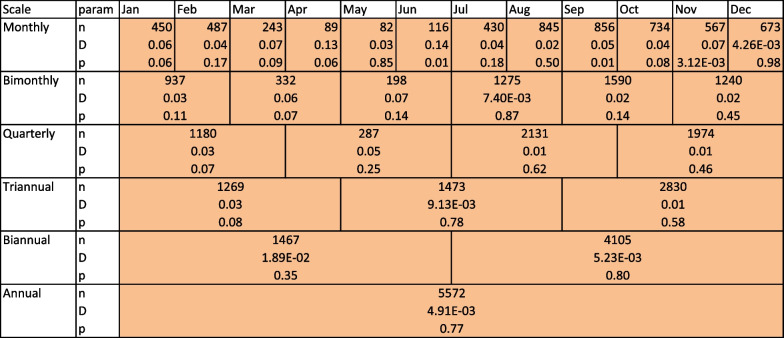
Summary of KS test results (selection for lower SST) for blue sharks at different aggregating scales

*n* refers to the sample size of observed blue sharks, *D* to the test statistic, and *p* to the p-value of the test. Cells are color-coded following the confidence in the test being correct, following the analysis of the previous section

## Discussion

In this analysis, we tested four different kinds of null movement models and showed how they could be used to test for environmental selection in marine animals, both with simulated synthetic tracks and observed blue shark tracks. The method presented here can be used to assess whether organisms select for specific conditions, provided with a large enough sample size and a strong enough selection (the sample size needed to assess selection decreases as the selection strength increases).

### Synthetic tracks analysis

Of the four null movement models we tested, Lévy walk has the highest rate of false positivity, and we caution its use as a null movement model (cf. Fig. 8B). In addition, many other types of null models exist and could be used for such study. For example, previous studies have simply rotated the entire track from the departure point, reshuffled the sampling dates [53], or estimated kernel densities and convex hulls [22, 31]. Variants of random walk models can also be developed, such as selecting random turning angles and drawing a step length not from a theoretical distribution (such as for Brownian motions and Lévy walks) but from empirical data [54], or creating reverse walks, i.e. starting the walk from the last known animal position and going backward in time [22]. Some of these methods are more appropriate than others depending on the study location or the question one tries to answer. For example, reshuffling dates and rotating tracks are more appropriate in land animal studies or when the animal does not wander too far off its home range, as this might otherwise lead to pseudo-absences in locations that are not accessible to the animal. A recent study found that when building habitat suitability models, the separation in environmental niche between presence and pseudo-absence was the most important driver of model explanatory power [22]. For blue whales tagged with ARGOS tags in the eastern North Pacific, this resulted in using background sampling, i.e. a random sampling of the entire study region.

Yet, random samplings of study locations may not be appropriate for all research questions, and different null models can be appropriate in different settings. In particular, Austin et al. [31] showed that home range indices (such as kernel home ranges) provide a single picture of the area occupied by an animal without considering the mechanisms and decisions that led the animal to move across the landscape. Using models based on complete spatial randomness may over-simplify the problem and lead to the “Jack Horner effect” [53, 55]—incorrectly rejecting the null hypothesis because the observed pattern is different from the null distribution because the null distribution is unreasonable. For example, a trivial null movement model would be comparing the SST of a basin scale migrator to the SST of a pseudo-animal that never moves far (e.g. a Brownian motion that is not periodically restarted). The fact that their SST distributions are significantly different is a trivial result based on global SST gradients, and not on environmental selection by the individual. At the other end of the spectrum, overly specified null models (e.g. null models restarted too frequently, for example every week or more) could mimic exactly the observed distribution, therefore leading to an incorrect validation of the null hypothesis – this is called the “Narcissus effect” [53]. To decrease the probability of drawing incorrect conclusions due to either of these effects, we recommend using an ensemble of null movement models [53, 56], as we have done here.

Another approach to habitat selection is step selection functions [23, 24, 26]. Step-selection approaches use a step (connecting two sequential observations) as the unit of measurement. The observed animal step is then compared to simulated steps, each time leaving from an observed animal position. This approach has several advantages, as it provides a way to simultaneously estimate parameters describing both movement and habitat selection processes [25, 26, 57], and as it can consider multiple environmental factors simultaneously and quantify complex selection patterns (e.g. quadratic effects where individuals select for medium values but avoid extremes—something that our approach relying on KS tests cannot do). It is possible to include random effects to account for correlation arising from consecutive steps (e.g. [58]), but all simulated random steps leave from an actual animal position, meaning that the available locations sampled will never be more than one step length away from the animal. This is adequate for fine-scale habitat selection, but it might bias medium- or large-scale habitat selection results, especially in marine organisms that routinely perform large relocation movements over several days to encounter more favourable conditions.

The strength of SST selection in synthetic tracks was determined by $\kappa$, i.e. the concentration of the Von Mises distribution relative to the direction of the highest SST within a 50 km radius. This metric is hard to relate to real animals as we do not know how strongly they might be selecting for SST or other environmental variables (and if we did there would not be a need for this study). Moreover, animals do not select for a single environmental factor such as SST, but likely combine different factors to increase their fitness. For example, oxygen concentration at depth for deep divers [59], or prey fields for large predators [60], are factors that are likely to influence and modify how an organism reacts to other environmental variables such as SST. Null models can, to some extent, help tease these effects apart and account for some of the known processes driving movement. For example, biased random walks can take into account the urge to migrate driven by external cues [61]. Current advection can be added to random walk for planktonic and small nektonic organisms. Memory, perception, potential environmental cues, and cost of locomotion can be implemented in random walk models [62, 63]. Taking into account these processes enables to ensure that they do not cloud potential results, thus helping to understand the importance of different environmental factors separately.

Finally, and as expected, selection results change when varying sample size and significance level. However, performing multiple tests increases the family-wise error rate (FWER), which is the probability of getting at least one false-positive in the series of tests performed. Here, for each selection of strength and sample size, we performed 100 different tests to get bootstrap estimates of our results. This explains in part why our rate of false-positive results is high. FWER can be kept at a pre-defined level by applying corrections such as a Bonferroni correction to the significance level (i.e. dividing the significance level by the number of tests performed). Here, we did not apply a Bonferroni correction as we knew the results to expect and wanted to test the strength of our method, and as future researchers willing to apply this method may not have enough data to perform multiple tests. Nevertheless, applying a Bonferroni correction does not change qualitatively our results (Additional file 1: Fig. S12), and the impact of modifying the significance level (which is essentially what a Bonferroni correction is) can be seen Figs. 10, S10, and S11.

### Observed blue shark tracks

Our case study (*n* = 57) concluded that blue sharks select (most of the time) for higher SST than environmentally naive organisms. The selection here is a “realized selection”, i.e. this selection emerges from the track but may not be the ultimate factor driving the movement. Based on this analysis, while we found that there was selection, we cannot ascertain the reasons why blue sharks select for higher SST (or other environmental parameters correlated with SST noting that, unlike synthetic organisms, blue sharks do not have a complete knowledge of SST gradients around them). For example, as most of the tags in this study were deployed in cooler foraging grounds of the California Current and animals tend to move south afterwards, our signal could be due to the species’ seasonal urge to migrate southward. Dividing the dataset in different temporal periods allows us to address that issue by investigating locations visited by the individuals several months after tagging—thus not biased by the spatio-temporal constraints associated with the tagging itself. In this particular case, as blue sharks are ectothermic animals using surface waters to warm themselves before diving down to depths of $\sim$400 m [64], it seems reasonable to hypothesize that blue sharks do indeed select for warmer SSTs for ecologically (prey distribution) or physiologically relevant reasons (thermoregulation, reproductive activities).

Testing hypothesis at the individual level is seldom possible because of the amount of data required to have statistically meaningful results. Aggregating data sets is an efficient way to increase sample size. Sequeira et al. [52] estimate that the identification of trait-specific behaviors and spatio-temporal patterns require between 10 and 100 different tracks (depending on tracks’ length). On the contrary, assessing spatio-temporal changes in habitat use or performing multispecies assessments at large spatial scales requires more than 100 tracked individuals. With this blue shark data set, more than $\sim$ 10 tracks correspond to an aggregation at the bimonthly to quarterly scale—scales at which results suggest that blue sharks select for higher SST, except in March-April.

Moreover, tracks, at least those based on ARGOS or GPS based tags, are biased by the (sometimes random or irregular) surfacing behavior of tagged individuals as ARGOS and GPS tags can only transmit data when individuals spend time at the surface. This has two main consequences. First, it means that our observations and results are biased towards the surface and that animals may in fact be selecting for specific environmental conditions deeper in the water column where they may spend more time. Second, it means that our knowledge of the position of individuals is sometimes imprecise, at times quite so (10’s to 100’s of kms). ARGOS data are generally processed using a state-space model [33] that interpolates between each data point (that already has some uncertainty, quantified by the location class of the ARGOS fix) to produce a track with a daily resolution. In addition to the location class of ARGOS fixes, confidence intervals of the processed track depends on the time interval between the processed data point and an ARGOS location fix. In our study, the SST used for locations with high uncertainties (and their corresponding pseudo-absences) is averaged over that large range of uncertainty. Consequently, the differences between presence SST and pseudo-absences SST will be minor, and we are unlikely to detect selection for that data point. Then, it appears that the confidence intervals (determined by the quality and frequency of raw data) will influence the rate of false-negative results, but not false-positive results (i.e. we detect selection less often than there actually is because the signal is “blurred” by the uncertainty). Increasing track accuracy may increase the rate of selection detected in individual tracks (but not much as most of these tracks are too short to confidently detect a signal), but it will modify results at the quarterly scale and above as we are already detecting selection at these scales. Investigating the influence of reporting quality and accuracy on environmental selection detection is an important venue for future research.

We investigated the differences between satellite-extracted and in-situ SST recorded by pop-up satellite archival tags (PAT or PSAT tags) for four blue sharks that were double-tagged with PAT and ARGOS tags. Even though results are very similar (see section C of the supplementary material and Additional file 1: Fig. S7), in-situ SST is higher than satellite SST when ARGOS tags are not transmitting data for a few days. The fact that ARGOS tags do not transmit data when sharks are experiencing higher SST might also indicate that the water is too warm for blue sharks there, or that blue sharks do not need to go all the way to the surface when the water column temperature is high enough, a behavior known as tropical submergence and previously reported in blue sharks [65]. Nevertheless, this consideration emphasizes that track accuracy and frequency of reporting is important to consider when testing for environmental selection in marine organisms. This could be tested further on animals tagged with both ARGOS and fast-loc GPS tags such as elephant seals. Elephant seals are ideal study animals for this question: as air breathers they need to surface regularly, and plenty of data already exist as they have been extensively tagged to monitor hydrographic conditions in the Southern Ocean [15].

## Conclusion

Given sufficient selection strength and sample size, the method presented here captures environmental selection of marine predators. This method can suffer from high false positive rates, especially in the absence of true selection. Our analysis with synthetic tracks quantifies the robustness of the method in different configurations.

KS tests as presented here only test whether organisms select for higher or lower values than environmentally naive organisms (represented by the four null movement models) would, but not if they select for particular SST values. Investigating the response and preference of individuals to a wide range of temperatures (and not selection for higher or lower temperatures) would further our understanding of the autecology of the studied species. The selection for optimal environmental conditions can be assessed with, for example, habitat selection or species distribution models [20]. Except for models that rely on count data rather than presence/absence, the same types of null models can be used in these modelling frameworks for generating pseudo absence data [21]. They can then further be used to provide predictions of habitat shifts in a changing climate [20].

A question that remains open are the spatial and temporal scales at which animals perform selection. In this study, our synthetic tracks selected higher temperatures at a daily scale, at a range of 50 km, on an 8 day average of SST values. These scales could have easily been changed for different synthetic tracks. For real animal tracks, the answer to this question is clouded by the scales at which data are acquired and at which our analysis is performed. But animals, especially large pelagic predators, may also select environmental conditions at a larger scale than 9 km. In this case, regridding satellite products at multiple spatio-temporal grains could help answer these questions.

## Supplementary Information

**Additional file 1.** Biased random walk simulation, additional analyses, and supplementary figures.

**Additional file 2.** Results of all KS tests performed on blue sharks.

## Data Availability

Code used to create null models and perform the case study analysis is available at https://gitlab.com/ud3/Environmental_selection. Null model tracks and their corresponding SST values are archived at the same address.

## Abbreviations

ARGOS: Advanced Research and Global Observation Satellite
CRW: Correlated Random Walk
CTD: Conductivity Temperature Depth (an electronic instrument that measures these properties)
GPS: Global Positioning System
JCRW: Joint Correlated Random Walk
KS: Kolmogorov-Smirnov
SPOT: Smart Positioning and Temperature (a tag providing ARGOS locations and temperature)
SST: Sea Surface Temperature
TOPP: Tagging of Pacific Predators
FWER: Family-Wise Error Rate

## References

[1] Walter GH, Hengeveld R (2000). The structure of the two ecological paradigms. Acta Biotheor.

[2] Rutz C, Hays GC (2009). New frontiers in biologging science. Biol Lett.

[3] Block BA, Jonsen ID, Jorgensen SJ, Winship AJ, Shaffer SA, Bograd SJ (2011). Tracking apex marine predator movements in a dynamic ocean. Nature.

[4] Hussey NE, Kessel ST, Aarestrup K, Cooke SJ, Cowley PD, Fisk AT (2015). Aquatic animal telemetry: a panoramic window into the underwater world. Science.

[5] Hays GC, Mortimer JA, Ierodiaconou D, Esteban N (2014). Use of long-distance migration patterns of an endangered species to inform conservation planning for the World’s Largest Marine Protected Area. Conserv Biol.

[6] Schorr GS, Falcone EA, Moretti DJ, Andrews RD. First long-term behavioral records from Cuvier’s beaked whales (*Ziphius cavirostris*) reveal record-breaking dives. PLoS ONE. 2014;9(3). 10.1371/journal.pone.0092633 PMC3966784 24670984

[7] Andrzejaczek S, Lucas TCD, Goodman MC, Hussey NE, Armstrong AJ, Carlisle A, et al. Diving into the vertical dimension of elasmobranch movement ecology. Science Advances. 2022;1–20. 10.1126/sciadv.abo1754 PMC9390984 35984887

[8] Quick NJ, Cioffi WR, Shearer JM, Fahlman A, Read AJ. Extreme diving in mammals: first estimates of behavioural aerobic dive limits in Cuvier’s beaked whales. J Exp Biol. 2020;223. 10.1242/jeb.222109 32967976

[9] Jorgensen SJ, Reeb CA, Chapple TK, Anderson S, Perle C, Van Sommeran SR (2010). Philopatry and migration of Pacific white sharks. Proc R Soc B: Biol Sci.

[10] Nosal AP, Cartamil DP, Ammann AJ, Bellquist LF, Ben-Aderet NJ, Blincow KM (2021). Triennial migration and philopatry in the critically endangered soupfin shark *Galeorhinus galeus*. J Appl Ecol.

[11] Benhamou S (2007). How many animals really do the Lévy walk?. Ecology.

[12] Nathan R, Getz WM, Revilla E, Holyoak M, Kadmon R, Saltz D (2008). A movement ecology paradigm for unifying organismal movement research. Proc Natl Acad Sci USA.

[13] Coffey DM, Carlisle AB, Hazen EL, Block BA (2017). Oceanographic drivers of the vertical distribution of a highly migratory, endothermic shark. Sci Rep.

[14] Miyazawa Y, Kuwano-Yoshida A, Doi T, Nishikawa H, Narazaki T, Fukuoka T, et al. Temperature profiling measurements by sea turtles improve ocean state estimation in the Kuroshio-Oyashio Confluence region. Ocean Dyn. 2019;69(2):267–282.

[15] Roquet F, Williams G, Hindell MA, Harcourt R, McMahon C, Guinet C (2014). A Southern Indian Ocean database of hydrographic profiles obtained with instrumented elephant seals. Sci Data.

[16] McMahon CR, Roquet F, Baudel S, Belbeoch M, Bestley S, Blight C (2021). Animal Borne Ocean Sensors - AniBOS - An Essential Component of the Global Ocean Observing System. Front Mar Sci.

[17] Braun CD, Gaube P, Sinclair-Taylor TH, Skomal GB, Thorrold SR (2019). Mesoscale eddies release pelagic sharks from thermal constraints to foraging in the ocean twilight zone. Proc Natl Acad Sci USA.

[18] Hazen EL, Carlisle AB, Wilson SG, Ganong JE, Castleton MR, Schallert RJ (2016). Quantifying overlap between the Deepwater Horizon oil spill and predicted bluefin tuna spawning habitat in the Gulf of Mexico. Sci Rep.

[19] Oliver MJ, Kohut JT, Bernard K, Fraser W, Winsor P, Statscewich H (2019). Central place foragers select ocean surface convergent features despite differing foraging strategies. Sci Rep.

[20] Hazen EL, Jorgensen S, Rykaczewski RR, Bograd SJ, Foley DG, Jonsen ID, et al. Predicted habitat shifts of Pacific top predators in a changing climate. Nature Clim Change. 2013;3(March).

[21] Aarts G, Fieberg J, Matthiopoulos J (2012). Comparative interpretation of count, presence-absence and point methods for species distribution models. Methods Ecol Evol.

[22] Hazen EL, Abrahms B, Brodie S, Carroll G, Welch H, Bograd SJ (2021). Where did they not go? Considerations for generating pseudo-absences for telemetry-based habitat models. Mov Ecol.

[23] Forester JD, Im HK, Rathouz PJ (2009). Accounting of resource selection functions: sampling and data analysis. Ecology.

[24] Thurfjell H, Ciuti S, Boyce MS (2014). Applications of step-selection functions in ecology and conservation. Mov Ecol.

[25] Duchesne T, Fortin D, Rivest LP (2015). Equivalence between step selection functions and biased correlated random walks for statistical inference on animal movement. PLoS ONE.

[26] Avgar T, Potts JR, Lewis MA, Boyce MS (2016). Integrated step selection analysis: bridging the gap between resource selection and animal movement. Methods Ecol Evol.

[27] Willis-Norton E, Hazen EL, Fossette S, Shillinger G, Rykaczewski RR, Foley DG (2015). Climate change impacts on leatherback turtle pelagic habitat in the Southeast Pacific. Deep-Sea Res II: Top Stud Oceanog.

[28] Wingfield DK, Peckham SH, Foley DG, Palacios DM, Lavaniegos BE, Durazo R (2011). The making of a productivity hotspot in the coastal ocean. PLoS ONE.

[29] Wakefield ED, Phillips RA, Trathan PN, Arata J, Gales R, Huin N (2011). Habitat preference, accessibility, and competition limit the global distribution of breeding Black-browed Albatrosses. Ecol Monogr.

[30] Raymond B, Lea MA, Patterson T, Andrews-Goff V, Sharples R, Charrassin JB (2015). Important marine habitat off east Antarctica revealed by two decades of multi-species predator tracking. Ecography.

[31] Austin D, Bowen WD, McMillan JI (2004). Intraspecific variation in movement patterns: modeling individual behaviour in a large marine predator. Oikos.

[32] Wisz MS, Guisan A. Do pseudo-absence selection strategies influence species distribution models and their predictions ? An information-theoretic approach based on simulated data. BMC Ecol. 2009;9(8). 10.1186/1472-6785-9-8 PMC2680809 19393082

[33] Jonsen I, Flemming JM, Myers R (2005). Robust state-space modeling of animal movement data. Ecology.

[34] Mardia KV, Jupp PE (1999). Directional statistics.

[35] JPL/OBPG/RSMAS. GHRSST Level 2P Global Sea Surface Skin Temperature from the Moderate Resolution Imaging Spectroradiometer (MODIS) on the NASA Aqua satellite (GDS2). MODIS Aqua L2P swath SST data set ver 20190. 2020.

[36] Chandrasekhar S (1943). Stochastic problems in physics and astronomy. Rev Mod Phys.

[37] Okubo A, Levin SA. Diffusion and Ecological Problems: Modern Perspectives. vol. 14 of Interdisciplinary Applied Mathematics. New York, NY: Springer; 2001. 10.1007/978-1-4757-4978-6.

[38] Codling EA, Plank MJ, Benhamou S (2008). Random walk models in biology. J R Soc Interface.

[39] Kranstauber B, Kays R, Lapoint SD, Wikelski M, Safi K (2012). A dynamic Brownian bridge movement model to estimate utilization distributions for heterogeneous animal movement. J Anim Ecol.

[40] Viswanathan GM, Buldyrev SV, Havlin S, da Luz MGE, Raposo EP, Stanley HE (1999). Optimizing the success of random searches. Nature.

[41] Bartumeus F, Catalan J, Fulco UL, Lyra ML, Viswanathan GM (2002). Optimizing the Encounter Rate in Biological Interactions: Lévy versus Brownian Strategies. Phys Rev Lett.

[42] Viswanathan GM, Afanasyev V, Buldyrev SV, Murphy EJ, Prince PA, Stanley HE (1996). Lévy flight search patterns of wandering albatrosses. Nature.

[43] Humphries NE, Queiroz N, Dyer JRM, Pade NG, Musyl MK, Schaefer KM (2010). Environmental context explains Lévy and Brownian movement patterns of marine predators. Nature..

[44] Bartumeus F, Peters F, Pueyo S, Marrasé C, Catalan J (2003). Helical Lévy walks: Adjusting searching statistics to resource availability in microzooplankton. Proc Natl Acad Sci USA.

[45] Edwards AM (2008). Using likelihood to test for Lévy flight search patterns and for general power-law distributions in nature. J Anim Ecol.

[46] James A, Plank MJ, Edwards AM (2011). Assessing Lévy walks as models of animal foraging. J R Soc Interface.

[47] Viswanathan GM, Raposo EP, da Luz MGE (2008). Lévy flights and superdiffusion in the context of biological encounters and random searches. Phys Life Rev.

[48] Bovet P, Benhamou S (1988). Spatial analysis of animals’ movements using a correlated random walk model. J Theor Biol.

[49] Bartumeus F, Da Luz MGE, Viswanathan GM, Catalan J (2005). Animal search strategies: a quantitative random-walk analysis. Ecology.

[50] Sequeira AMM, Rodríguez JP, Eguíluz VM, Harcourt R, Hindell M, Sims DW (2018). Convergence of marine megafauna movement patterns in coastal and open oceans. Proc Natl Acad Sci USA.

[51] Hodel FH, Fieberg JR (2022). Circular-linear copulae for animal movement data. Methods Ecol Evol.

[52] Sequeira AMM, Heupel MR, Lea MA, Eguíluz VM, Duarte CM, Meekan MG (2019). The importance of sample size in marine megafauna tagging studies. Ecol Appl.

[53] Miller JA (2015). Towards a better understanding of dynamic interaction metrics for wildlife: a null model approach. Trans GIS.

[54] Sims DW, Witt MJ, Richardson AJ, Southall EJ, Metcalfe JD (2006). Encounter success of free-ranging marine predator movements across a dynamic prey landscape. Proc R Soc B: Biol Sci.

[55] Wilson JB (1995). Null models for assembly rules: The Jack Horner effect is more insidious than the narcissus effect. Oikos.

[56] Dale MRT, Fortin MJ (2014). Spatial analysis: a guide for ecologists.

[57] Fieberg J, Signer J, Smith B, Avgar T (2021). A ‘How to’ guide for interpreting parameters in habitat-selection analyses. J Anim Ecol.

[58] Muff S, Signer J, Fieberg J (2020). Accounting for individual-specific variation in habitat-selection studies: efficient estimation of mixed-effects models using Bayesian or frequentist computation. J Anim Ecol.

[59] Stramma L, Prince ED, Schmidtko S, Luo J, Hoolihan JP, Visbeck M (2012). Expansion of oxygen minimum zones may reduce available habitat for tropical pelagic fishes. Nat Clim Chang.

[60] Benoit-Bird KJ, Battaile BC, Heppell SA, Hoover B, Irons D, Jones N, et al. Prey patch patterns predict habitat use by top marine predators with diverse foraging strategies. PLoS ONE. 2013;8(1). 10.1371/journal.pone.0053348 PMC3536749 23301063

[61] Bailey JD, Wallis J, Codling EA (2018). Navigational efficiency in a biased and correlated random walk model of individual animal movement. Ecology.

[62] Fronhofer EA, Hovestadt T, Poethke HJ (2013). From random walks to informed movement. Oikos.

[63] Pinti J, Celani A, Thygesen UH, Mariani P (2020). Optimal navigation and behavioural traits in oceanic migrations. Theoretical Ecology.

[64] Watanabe YY, Nakamura I, Chiang WC (2021). Behavioural thermoregulation linked to foraging in blue sharks. Marine Biol.

[65] Stevens JD, Bradford RW, West GJ (2010). Satellite tagging of blue sharks (Prionace glauca) and other pelagic sharks off eastern Australia: depth behaviour, temperature experience and movements. Mar Biol.

