# Supplementary material for: Using pseudo-absence models to test for environmental selection in marine movement ecology: the importance of sample size and selection strength
Source: Mov Ecol. 2022 Dec 29;10:60. doi: 10.1186/s40462-022-00362-1 (PMC9798696; doi:10.1186/s40462-022-00362-1)
Supplement: Supplementary file 1 — Additional file 1. Biased random walk simulation, additional analyses, and supplementary figures. [file 40462_2022_362_MOESM1_ESM.pdf]

Supporting Information 1 for "Using pseudo-absence models to test for environmental selection in marine movement ecology: the importance of sample size and selection strength"

Jérôme Pinti[1], Matthew Shatley[1], Aaron Carlisle[1], Barbara A. Block[2], Matthew J. Oliver[1]

[1] College of Earth, Ocean, and Environment, University of Delaware, Lewes, DE 19958, USA

[2] Hopkins Marine Station, Biology Department, Stanford University, Pacific Grove, CA 93950, USA

A Biased random walk simulation

For different strengths of temperature selection, 100 synthetic tracks were produced using biased random walks. Each track is 80 days long. Step length was fixed at 50 km per day, and bearing (not turning angle) was generated following a Von Mises probability distribution with concentration κ. The distribution is centered at the direction corresponding to that of the highest SST within a 50km radius, and κ indicates how concentrated the probability distribution is around that direction.

SST was acquired from MODIS-AQUA environmental products at a daily resolution of 9km, and averaged over 8 days to decrease data patchiness. As organisms cannot know the temperature of the following days, the average performed was a backward rolling average (i.e., for each day x, the average was realized using SST from days x, $x-1$, $x-2$, ..., $x-7$).

For each of the synthetic tracks, the departure location was randomly selected in the North East Pacific between 10 and 40 °N and 130 and 140 °E. The departure date is May 1st 2006 for all tracks. Simulated tracks for different strengths of temperature selection can be seen figure S1.

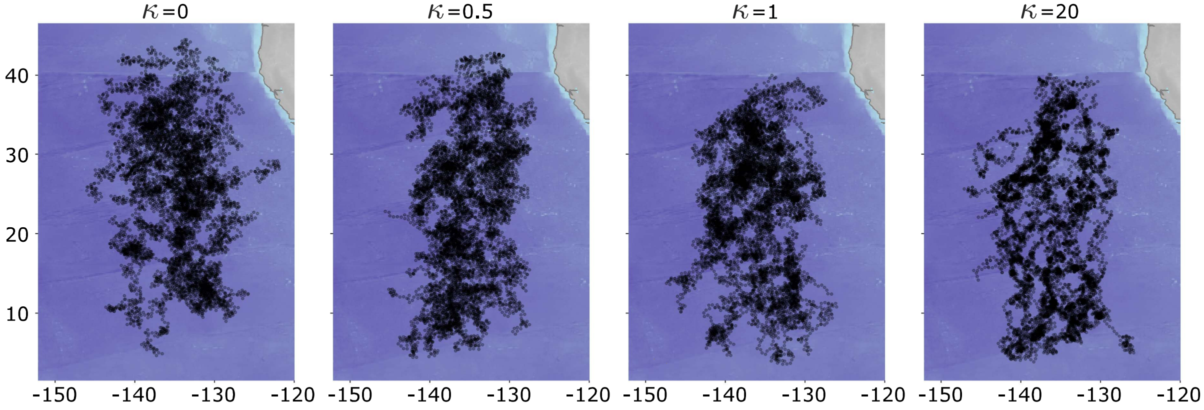

Figure S1: 100 simulated biased random walks for different strengths of temperature selection.

B Importance of track reinitialization

The frequency at which tracks are restarted directly impacts the rate of false-positive and false-negative rates. We investigate this specifically for Brownian motion as null models here, but the results are qualitatively the same for

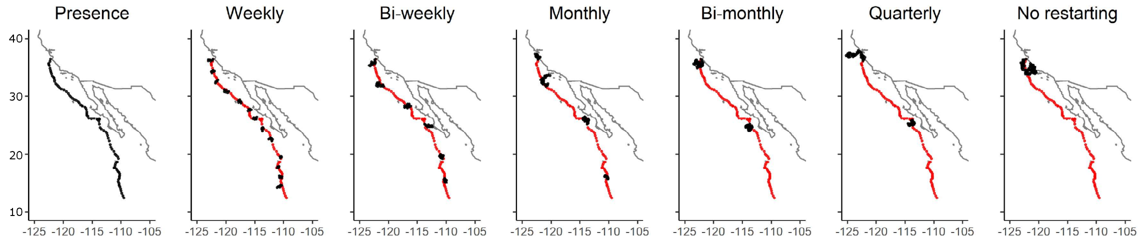

Figure S2: Track #160401201 and pseudo-absence tracks (Brownian motion) with different restarting frequencies. Pseudo-absences are overlaid on top of presence observations.

the other null models. Increasing the restarting frequency increases how close pseudo-absences are from presence observations (figures S2, S3 and S4). Consequently, this increases how similar SST presence and pseudo-absences are (figures S5 and S6). Increasing the restarting frequency then decreases our ability to detect environmental selection (and thus increases the rate of false-negative results), as presence and pseudo-absence SST distributions are very similar (figure S6, and tables S1, S2, S3 and S4). At the quarterly scale (i.e. aggregating the data by quarters), only the 4th quarter leads to a significant result for KS-tests performed on pseudo-absences with a weekly restarting frequency, whereas all tests performed with a monthly or bi-monthly restarting frequency are significant (table S3).

Conversely, decreasing the restarting frequency increases the detection of selection (and thus the rate of false-positive results) as pseudo-absences do not take into account large scale movement of organisms. At the monthly scale, not restarting the null model or restarting them only quarterly leads to detecting selection for higher SST all months of the year – except in April, where only 89 data points are available (table S1).

The choice of the restarting frequency depends on how conservative one wants to be regarding false-positive and false-negative rates. The more often the tracks are reset (i.e. the higher the restarting frequency), the more conservative the results are, i.e. the lower the rate of false-positive results. For this analysis, we decided to restart all our tracks monthly, to be able to detect selection while minimizing both the rates of false-negative and false-positive results.

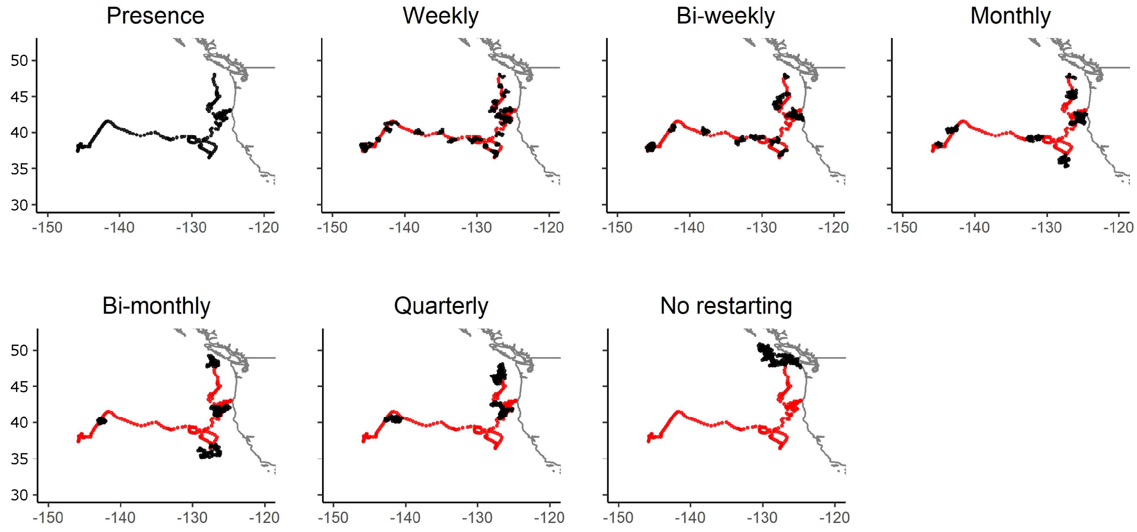

Figure S3: Track #160700501 and pseudo-absence tracks (Brownian motion) with different restarting frequencies. Pseudo-absences are overlaid on top of presence observations.

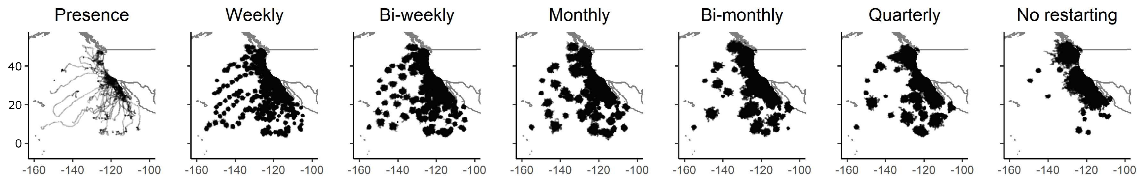

Figure S4: Blue shark tracks and associated pseudo-absences (Brownian motion) with different restarting frequencies.

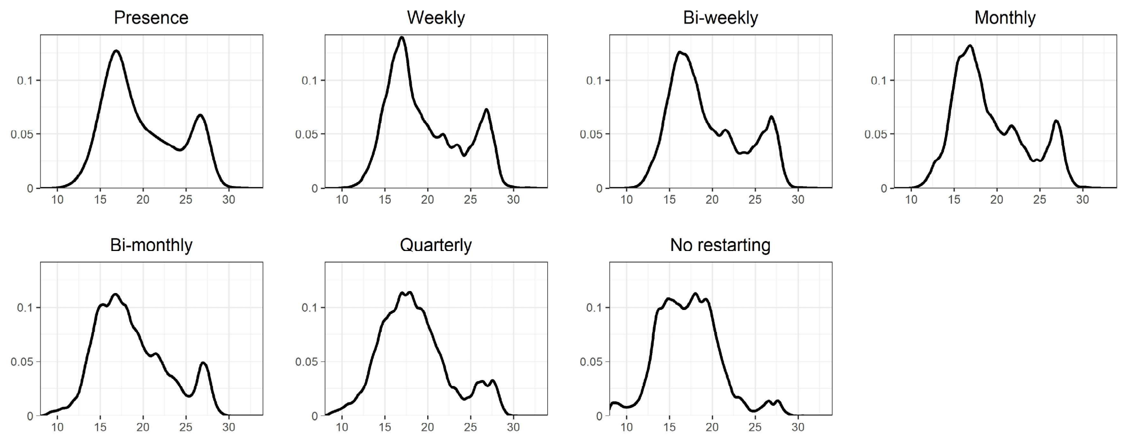

Figure S5: SST distribution for presence and pseudo-absences (Brownian motion) with different restarting frequencies.

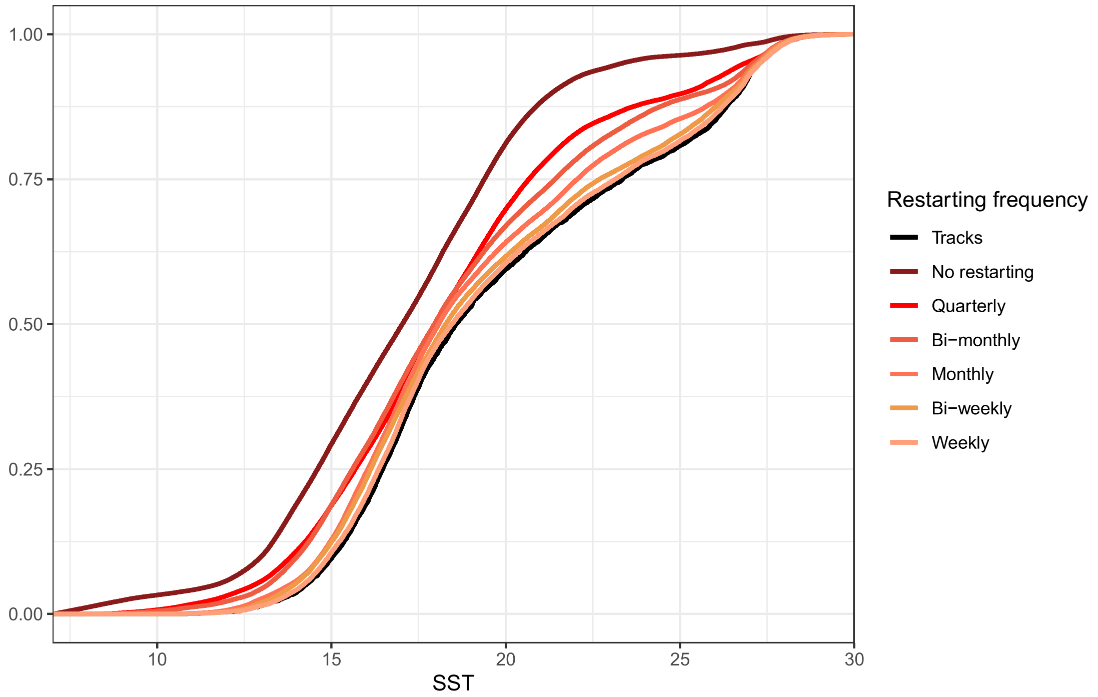

Figure S6: Cumulative SST distribution for presence and pseudo-absences (Brownian motion) with different restarting frequencies.

Table S1: Summary of KS test results (selection for higher SST) for blue sharks data aggregated at the monthly scale, when restarting the null models at different frequencies. D is the test statistic, and p the p-value of the test. Cells are color-coded in green for p-values below 0.01, in light red for p-values above 0.05, and in grey for p-values between 0.01 and 0.05.

Scale	param	Jan	Feb	Mar	Apr	May	Jun	Jul	Aug	Sep	Oct	Nov	Dec
No restarting	D	0.61	0.35	0.26	0.085	0.25	0.40	0.13	0.17	0.32	0.45	0.28	0.60
	p	1.8E-157	1.1E-63	3.5E-20	0.20	8.7E-06	2.5E-16	6.1E-08	1.7E-23	1.2E-76	1.9E-127	9.7E-40	6.6E-231
Quarterly	D	0.23	0.21	0.14	0.040	0.29	0.51	0.07	0.16	0.32	0.10	0.25	0.57
	p	4.9E-23	3.0E-22	1.2E-06	0.73	1.3E-07	4.7E-27	1.8E-02	1.1E-19	5.6E-77	4.8E-07	1.2E-32	7.4E-208
Bimonthly	D	0.23	0.20	0.04	0.08	0.29	0.53	0.07	0.16	0.07	0.16	0.21	0.56
	p	4.2E-23	5.4E-21	0.32	0.29	1.0E-07	2.1E-28	9.6E-03	1.8E-19	5.4E-05	2.3E-16	1.7E-23	1.1E-200
Monthly	D	0.21	0.15	0.040	0.040	0.29	0.32	0.070	0.12	0.074	0.10	0.21	0.32
	p	3.9E-22	2.5E-11	0.33	0.64	1.0E-07	4.9E-06	0.011	1.0E-10	6.4E-05	6.4E-07	3.7E-23	9.4E-65
Bi-weekly	D	0.10	0.07	0.05	0.08	0.15	0.14	0.081	0.043	0.039	0.07	0.18	0.14
	p	6.8E-05	3.0E-03	0.20	0.24	0.014	0.009	2.5E-03	0.039	0.069	2.1E-03	1.1E-16	2.1E-13
Weekly	D	0.06	0.04	0.04	0.03	0.07	0.05	0.04	0.03	0.03	0.03	0.08	0.07
	p	0.039	0.10	0.33	0.81	0.41	0.53	0.18	0.32	0.29	0.38	8.8E-04	6.5E-04

Table S2: Summary of KS test results (selection for higher SST) for blue sharks data aggregated at the bi-monthly scale, when restarting the null models at different frequencies. D is the test statistic, and p the p-value of the test. Cells are color-coded in green for p-values below 0.01, in light red for p-values above 0.05, and in grey for p-values between 0.01 and 0.05.

Scale	param	Jan-Feb	Mar-Apr	May-Jun	Jul-Aug	Sep-Oct	Nov-Dec
No restarting	D	0.47	0.21	0.32	0.14	0.36	0.46
	p	2.5E-202	2.2E-17	2.6E-19	4.9E-23	2.5E-181	5.3E-238
Quarterly	D	0.21	0.11	0.40	0.11	0.22	0.43
	p	6.5E-40	3.0E-05	1.4E-30	2.0E-14	1.3E-65	1.5E-210
Bimonthly	D	0.20	0.029	0.41	0.11	0.10	0.40
	p	3.5E-39	0.46	7.9E-32	3.8E-14	1.9E-14	4.9E-187
Monthly	D	0.12	0.029	0.25	0.075	0.072	0.23
	p	6.0E-15	0.46	2.2E-12	3.1E-07	5.9E-08	6.0E-63
Bi-weekly	D	0.067	0.035	0.12	0.035	0.045	0.14
	p	6.3E-05	0.340	1.6E-03	0.037	0.0015	1.3E-22
Weekly	D	0.032	0.032	0.047	0.019	0.016	0.060
	p	0.12	0.40	0.40	0.40	0.43	7.8E-05

Table S3: Summary of KS test results (selection for higher SST) for blue sharks data aggregated at the quarterly scale, when restarting the null models at different frequencies. D is the test statistic, and p the p-value of the test. Cells are color-coded in green for p-values below 0.01, in light red for p-values above 0.05, and in grey for p-values between 0.01 and 0.05.

Scale	param	Jan-Feb-Mar	Apr-May-Jun	Jul-Aug-Sep	Oct-Nov-Dec
No restarting	D	0.41	0.20	0.21	0.33
	p	4.0E-210	7.8E-12	1.8E-84	1.7E-192
Quarterly	D	0.19	0.27	0.19	0.30
	p	4.0E-44	8.8E-21	1.7E-70	1.3E-159
Bimonthly	D	0.16	0.27	0.09	0.29
	p	3.1E-30	3.0E-21	6.7E-17	1.4E-148
Monthly	D	0.09	0.17	0.07	0.16
	p	1.1E-11	8.9E-09	2.0E-10	8.2E-49
Bi-weekly	D	0.05	0.09	0.03	0.10
	p	5.5E-04	7.7E-03	0.016	1.2E-18
Weekly	D	0.027	0.031	0.013	0.044
	p	0.13	0.52	0.46	4.2E-04

Table S4: Summary of KS test results (selection for higher SST) for all blue sharks observations, when restarting the null models at different frequencies. D is the test statistic, and p the p-value of the test. Cells are color-coded in green for p-values below 0.01, in light red for p-values above 0.05, and in grey for p-values between 0.01 and 0.05.

Scale	param	Year
No restarting	D	0.23
	p	1.2E-288
Quarterly	D	0.13
	p	1.5E-93
Bimonthly	D	0.10
	p	6.7E-53
Monthly	D	0.059
	p	1.0E-18
Bi-weekly	D	0.04
	p	5.7E-11
Weekly	D	0.02
	p	0.048

C Double-tagged blue sharks

Four blue sharks were double-tagged with SPOT and pop-up archival tags. Pop-up archival tags record temperature and pressure of organisms, which allows us to compute directly the average SST that an individual experienced daily. We compared the satellite-based SST computed from tracks processed by the state-space model and the average daily SST encountered by these double-tagged blue sharks for the period where the two tags recorded data (figure S7). For shark #160400301, this period lasted 101 days, over which 110 ARGOS locations (with location class ≥ 0) were recorded in 47 different days (figure S7A, B). Shark #160402201 had both its tags working simultaneously for 104 days, over which 51 ARGOS locations (with location class ≥ 0) were recorded in 46 different days (figure S7C, D). Shark #160400801 had both its tags working simultaneously for 40 days, over which 133 ARGOS locations (with location class ≥ 0) were recorded in 30 different days (figure S7E, F). Shark #160601501 had both its tags working simultaneously for 79 days, over which 107 ARGOS locations (with location class ≥ 0) were recorded in 40 different days (figure S7G, H).

Sharks with lots of raw ARGOS locations relative to track length (e.g. 160400301, 160400801) have quite similar distributions (figure S7A and E), while sharks with scarcer records (e.g. 160402201) have distributions that do not overlap as well (panel C). As expected, the largest differences between satellite and in-situ SST appear when no ARGOS data are transmitted for several days (see e.g. panel D and F).

Whether these two SST distributions are different can be tested with two-sided Kolmogorov-Smirnov tests. No significant difference in SST distribution was detected for sharks 160400301 and 160400801 (p-value of 0.42 and 0.40, respectively), and the difference in SST distribution for shark 160600501 was not significant at the 0.01 level (p-value of 0.018). The difference in SST distribution for shark 160402201 is significant (p-value of $6.2 \cdot 10^{-4}$). It is noteworthy that shark 160402201 is the shark with the lowest number of ARGOS hits relative to track length of the three double-tagged sharks, with less than 1 location per tracking day on average (51 ARGOS locations for a track of 104 days).

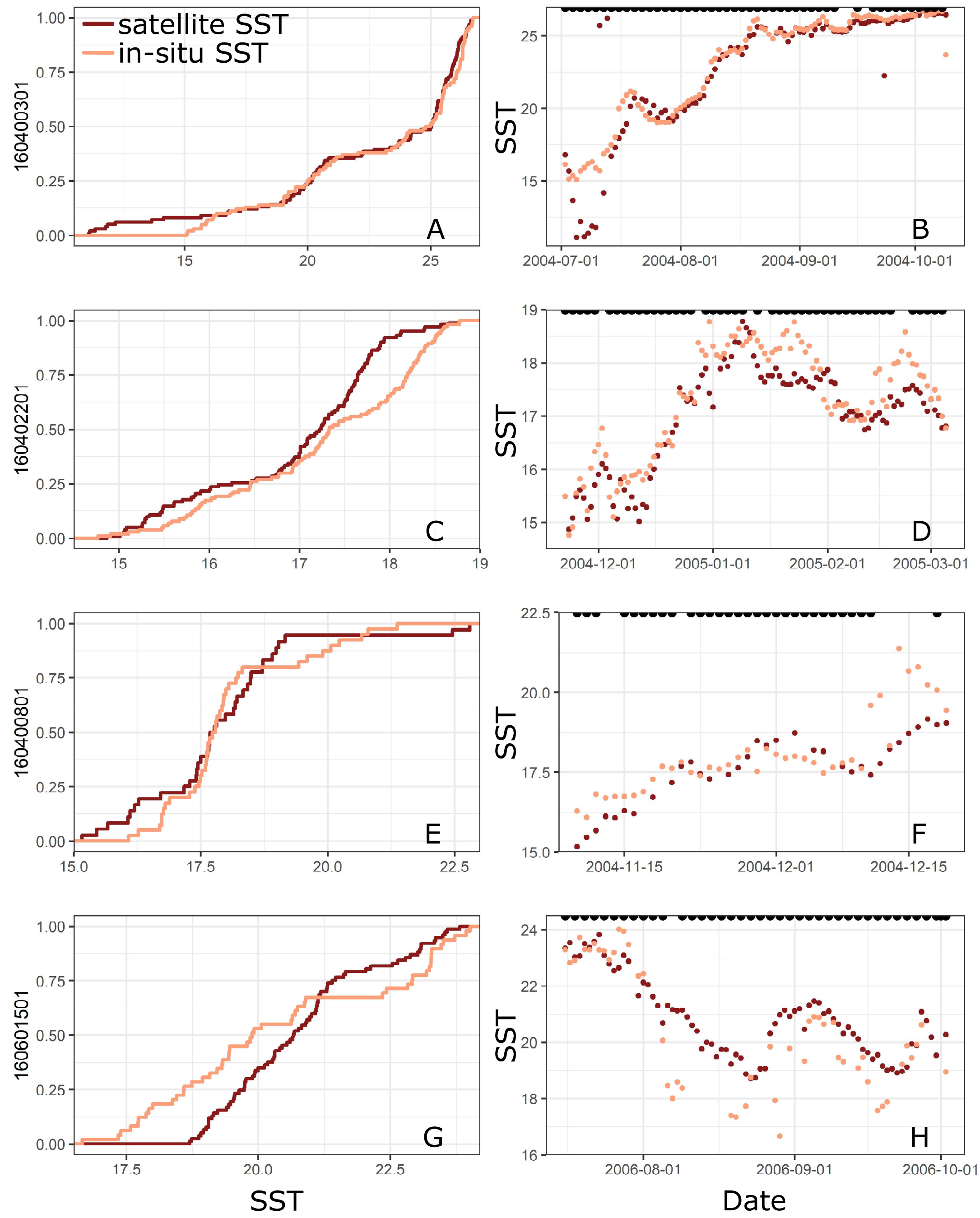

Figure S7: Comparison of satellite SST extracted from state-space tracks and in-situ SST from pop-up satellite archival tags for four double-tagged blue sharks. Left column: Cumulative SST. Right column: SST as a function of time. Black dots on top of panels of the right columns indicate days where ARGOS data were acquired with SPOT tags.

D Supplementary figures

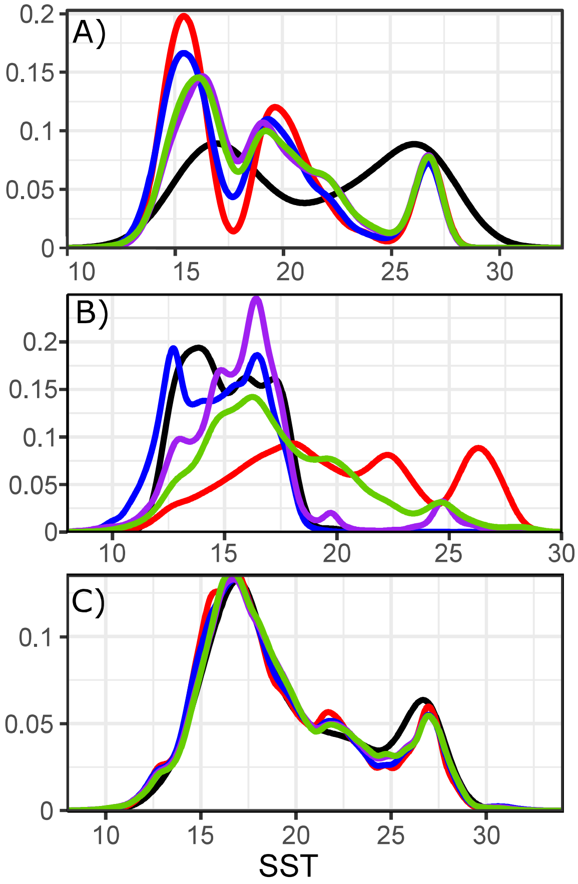

Figure S8: Density distribution of SST encountered by blue sharks for (A) shark #160401201 alone, (B) shark #160700501 alone, and (C) for all tagged blue sharks. Black lines represent tagged animals (presence), while red, blue, purple, and green represent pseudo-absence data (simulated with Brownian motion, Lévy walks, Correlated Random Walk, and Joint Correlated Random Walk, respectively.)

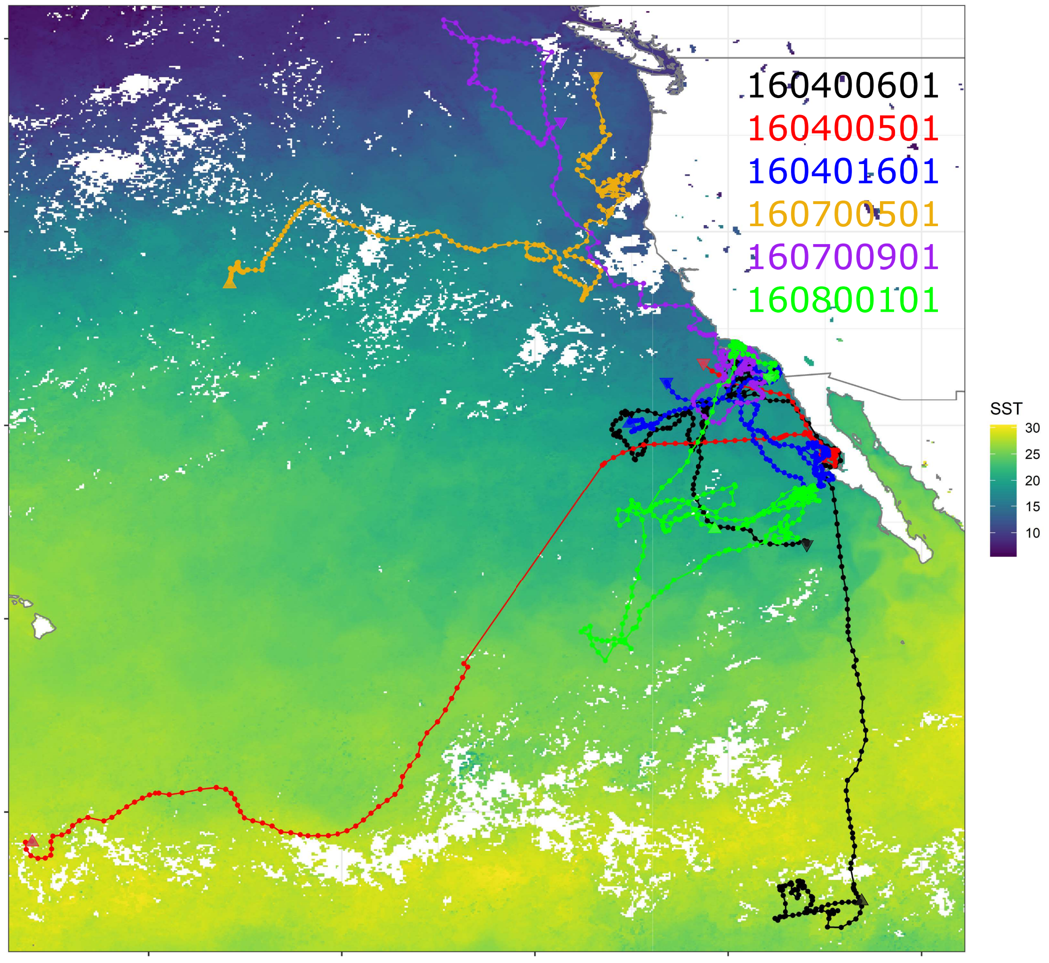

Figure S9: The six longest blue shark tracks of the dataset. Tracks are overlaid on top of SST field at the date November 15th 2004. The gap in the track of shark #160400501 is because no ARGOS data was acquired for more than 20 days, meaning that the SSM did not interpolate between the two locations. Consequently, no analysis was performed using that section of the track with no data.

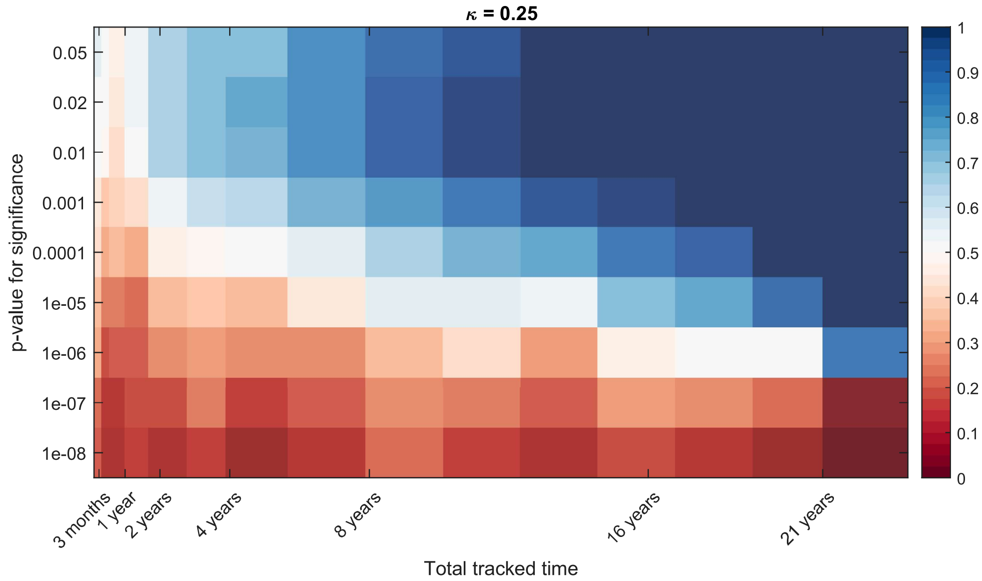

Figure S10: Fraction of significant test results when testing for higher SST as a function of data set size and p-value threshold for significance, when $\kappa = 0.25$ (weak SST selection). Blue values indicate a high fraction of correct outcomes, while red values indicate that no selection was detected (false negative result).

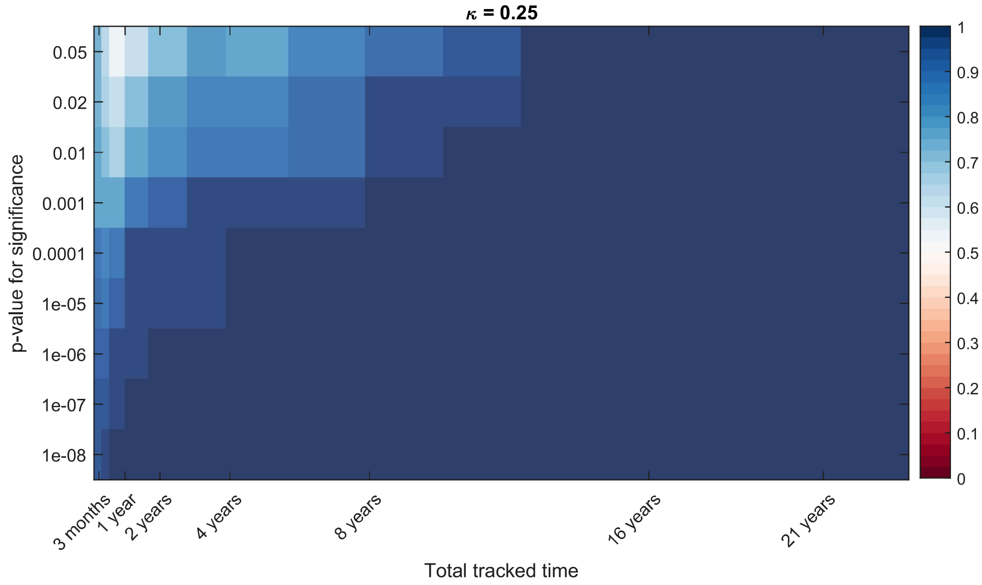

Figure S11: Fraction of **non** significant test results when testing for selection for lower SST, as a function of data set size and p-value threshold for significance, when $\kappa = 0.25$ (weak SST selection). Blue values indicate a high fraction of correct outcomes, while red values indicate that selection for lower SST was detected (false positive result).

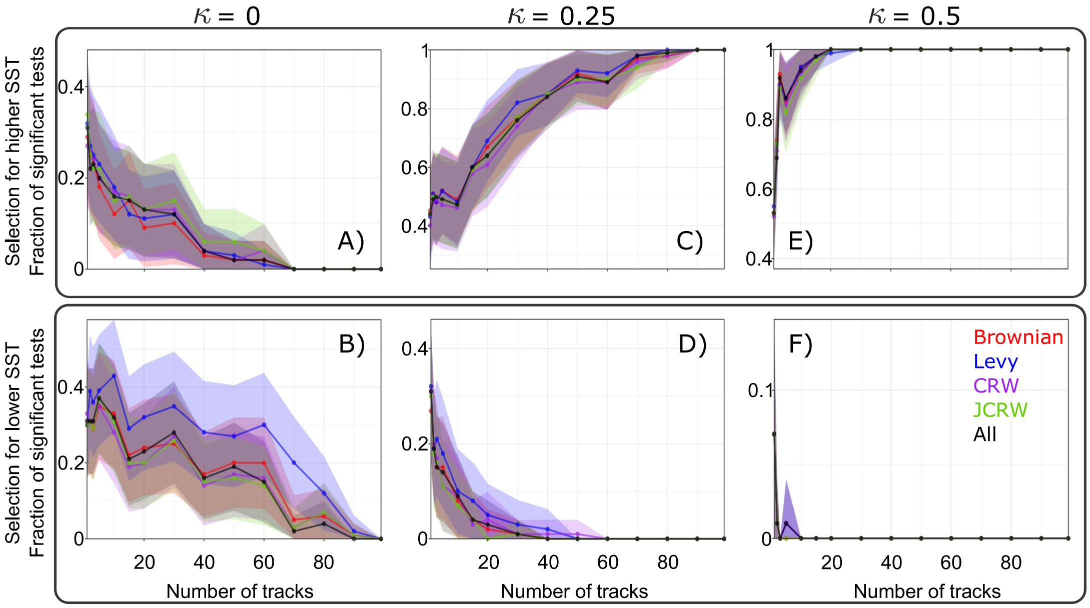

Figure S12: Fraction of significant test results as a function of the number of tracks considered for different values of κ, along with bootstrapped 95% confidence intervals. Tests performed with a Bonferroni correction for a p-value of 0.05 (so with a corrected significance level of $5 \cdot 10^{-4}$ as we are performing 100 tests each time). Top (A, C, E): Tests for higher SST selection. Bottom (B, D, F): Tests for lower SST selection.
